# Surface Immobilization of Human Arginase-1 with an Engineered Ice Nucleation Protein Display System in *E*. *coli*

**DOI:** 10.1371/journal.pone.0160367

**Published:** 2016-08-01

**Authors:** Zhen Zhang, Rongxin Tang, Lu Bian, Meng Mei, Chunhua Li, Xiangdong Ma, Li Yi, Lixin Ma

**Affiliations:** 1 Hubei Collaborative Innovation Center for Green Transformation of Bio-resources, Hubei Key Laboratory of Industrial Biotechnology, College of Life Sciences, Hubei University, Wuhan, People’s Republic of China; 2 Beihai Blood Center, Guang Xi, People’s Republic of China; Imperial College London, UNITED KINGDOM

## Abstract

Ice nucleation protein (INP) is frequently used as a surface anchor for protein display in gram-negative bacteria. Here, MalE and TorA signal peptides, and three charged polypeptides, 6×Lys, 6×Glu and 6×Asp, were anchored to the N-terminus of truncated INP (InaK-N) to improve its surface display efficiency for human Arginase1 (ARG1). Our results indicated that the TorA signal peptide increased the surface translocation of non-protein fused InaK-N and human ARG1 fused InaK-N (InaK-N/ARG1) by 80.7% and 122.4%, respectively. Comparably, the MalE signal peptide decreased the display efficiencies of both the non-protein fused InaK-N and InaK-N/ARG1. Our results also suggested that the 6×Lys polypeptide significantly increased the surface display efficiency of K_6_-InaK-N/ARG1 by almost 2-fold, while also practically abolishing the surface translocation of non-protein fused InaK-N, indicating the interesting roles of charged polypeptides in bacteria surface display systems. Cell surface-immobilized K_6_-InaK-N/ARG1 presented an arginase activity of 10.7 U/OD_600_ under the optimized conditions of 40°C, pH 10.0 and 1 mM Mn^2+^, which could convert more than 95% of L-Arginine (L-Arg) to L-Ornithine (L-Orn) in 16 hours. The engineered InaK-Ns expanded the INP surface display system, which aided in the surface immobilization of human ARG1 in *E*. *coli* cells.

## Introduction

Bacterial surface display has been widely applied in scientific research and the science industry [1]. Various functional peptides, recombinant vaccines, and catalytic enzymes could be displayed on bacterial surfaces through appropriate surface anchors, which facilitated their further engineering and applications for certain purposes [2]. In gram-negative bacteria, two major secretion systems, the general secretion (Sec) and twin arginine translocation (Tat) pathways [3–4], have been identified to transport proteins to the extracellular milieu under the guidance of various N-terminal signal peptides. Most surface carriers in bacteria are surface presenting proteins that use an N-terminal signal sequence to guide their translocation to the cell surface, including OmpA, Omp85, and LamB [5–6]. By contrast, INPs represented a special type of surface carriers with unidentified signal sequences [7–8]. Interestingly, full-length INP as well as its truncated forms could both be presented on the cell surface with the GPI anchor [9].

**Fig 1:**
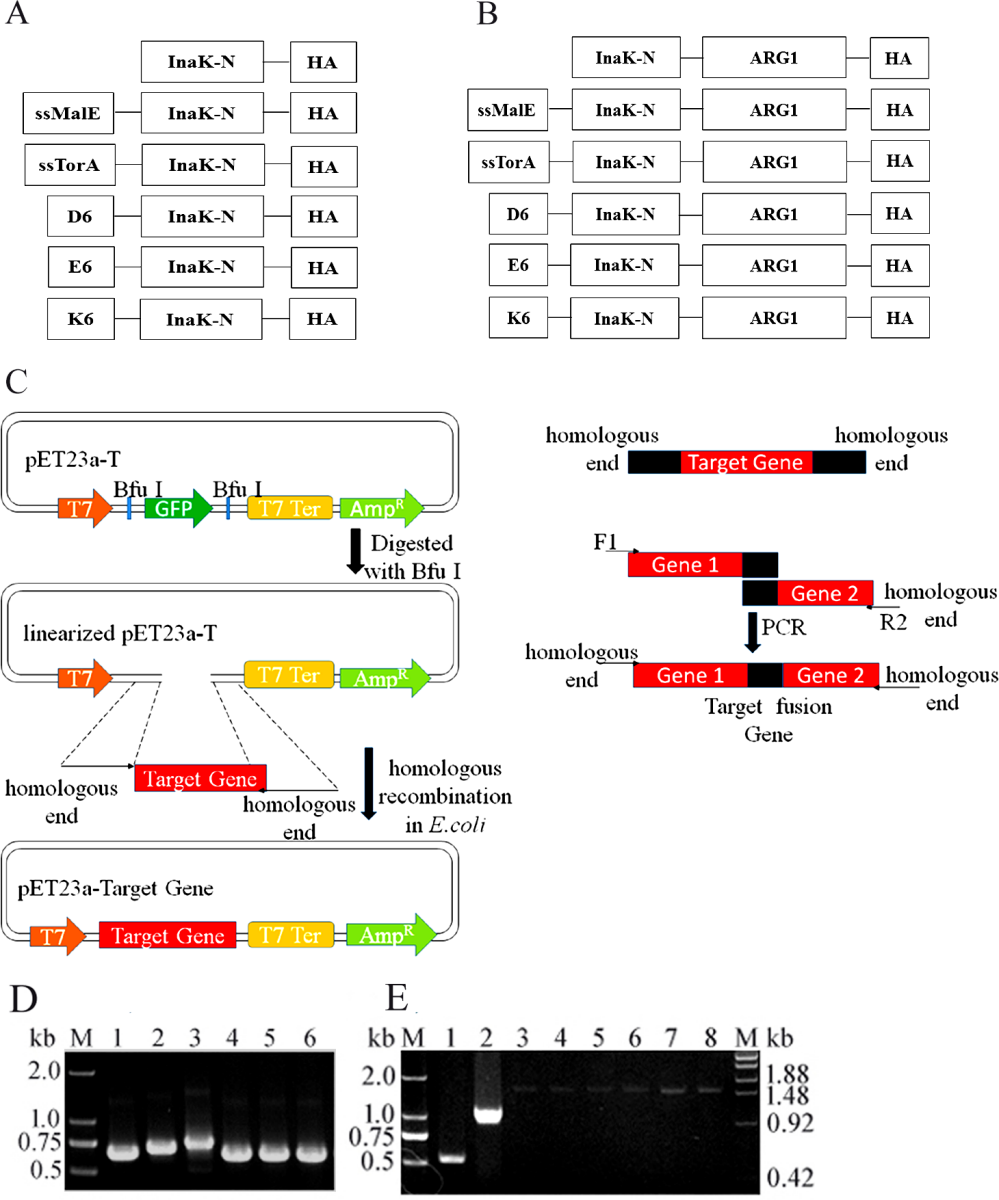
Constructs in the study. (A) Different N-terminal fusions of InaK-N. (B) Different InaK-Ns fused with C-terminal human ARG1. (C) Schematic diagram of the cloning method for making the constructs. (D) *InaK-Ns* PCR products. Lanes 1–6 are PCR products for *InaK-N*, *ssMalE*-*InaK-N*, *ssTorA*-*InaK-N*, *D*_*6*_-*InaK-N*, *E*_*6*_-*InaK-N*, and *K*_*6*_-*InaK-N*. (E) PCR products for *InaK-N/ARG1s*. Lane 1–8 *InaK-N*, *human ARG1*, *InaK-N/ARG1*, *ssMalE*-*InaK/ARG1*, *ssTorA*-*InaK-N/ARG1*, *D*_*6*_-*InaK-N/ARG1*, *E*_*6*_-*InaK-N/ARG1*, and *K*_*6*_-*InaK-N/ARG1*. M: DNA Ladder.

Here, we reported optimization of a bacterial INP surface display system. Truncated INP (InaK-N), which only contains its N-terminal region, was engineered by adding different polypeptides to its N-terminus to improve its surface display efficiency (Table 1). Briefly, two signaling peptides, MalE and TorA, and three charged polypeptides, 6×Lys, 6×Glu, and 6×Asp, were added to the N-terminal of InaK-N, forming newly constructed surface anchors, in which the foreign protein could be fused at the C-terminus of InaK-N for cell surface immobilization (Fig 1). Using this optimized surface display system, human ARG1, an enzyme that catalyzes the hydroxylation of L-Arg to L-Orn and urea, was successfully displayed on *E*. *coli* cell surfaces. Human ARG1 was crucial to human health, and its deficiency was the main cause of developmental delay, intellectual disability, seizures and ataxia [10]. More interestingly, its application has been expanded to cancer therapeutics by depleting arginine content in cancer cells [10–11]. However, because human ARG1 has to form a trimer to perform its function, it was difficult to actively immobilize the enzyme on the cell surface, which limited its further engineering using surface display-mediated high-throughput methods.

**Table 1:** Sequences of the export signals and polypeptides used in this study.

Signal peptide/Polypeptide	Sequence^a^
MalE	MKIKTGARILALSALTTMMFSASALA
TorA	MNNNDLFQASRRRFLAQLGGLTVAGMLGPSLLTPRRATAAQAA
D6	DDDDDD
K6	KKKKKK
E6	EEEEEE
InaK-N	MVLDKALVLRTCANNMADHCGLIWPASGTVESRYWQSTRRHENGLVGLLWGAGTSAFLSVHADARWIVCEVAVADIISLEEPGMVKFPRAEVVHVGDRISASHFISARQADPASTSTSTSTSTLTPMPTAIPTPMPAVASVTLPVAEQARHEVFDVASVSAAAAPVNTLPVTTPQNLQT

^a^ The sequence is given in the N- to C-terminal direction.

Our results indicated that the N-terminal TorA signal peptide as well as 6×Lys and 6×Glu could significantly prompt the surface immobilization of human ARG1. The N-terminal TorA signal peptide increases the cell surface display efficiency of InaK-N by 80.7%. Meanwhile, N-terminal 6×Lys polypeptides exhibited an approximately 2-fold up-regulated effect on K_6_-InaK-N/ARG1 surface translocation. Further characterization of the surface-immobilized K_6_-InaK-N/ARG1 demonstrated that it could almost fully hydrolyze L-Arg to L-Orn in 16 hours, displaying an arginase activity of 10.7 U/OD_600_. To the best of our knowledge, the work presented here was the first report that human ARG1 could be actively displayed on the *E*. *coli* surface through an INP display system. More importantly, as there are broad interests in ARG1 for industry and cancer therapy [10–11], the findings from our study provide alternative methods for its application and engineering. Additionally, our research also suggests new ideas for surface immobilization and the further engineering of complex proteins in *E*. *coli*.

## Materials and Methods

### Vector construction and protein induction

The *InaK-N* gene from *P*.*syringae* (KCTC1832) with a HA epitope tag sequence was synthesized based on *E*. *coli* codon usage bias [12]. Then, the different polypeptide DNA sequences, including MalE, TorA, 6×Lys, 6×Glu, and 6×Asp (Table 1), were added to the 5’ end of *InaK-N* (Fig 1A, Table 1). The human ARG1 gene was amplified from a previous work [13] and fused to the different InaK-Ns (Fig 1A and 1B). All of the DNA fragments were cloned into the pET23a-T vector [14] for protein expression (Fig 1C).

*Rosetta Blue E*.*coli* cells (Novagen, USA) harboring different constructs were expressed. Each singe clone was picked and inoculated in 100 mL of LB media with 50 μg/ml ampicillin till OD600 value between 0.5 and 0.6, IPTG was then added into the media at final concentration 1mM followed by 37°C for another 8 hours at 200 rpm.

### Outer-membrane protein extract and analysis

After cell induction, cells were harvest, and outer membrane proteins were enriched according to a rapid isolation method [15]. Protein samples were analyzed using SDS-PAGE (12% gels) and followed by western blot against the HA epitope tag.

### Proteinase K accessibility assay

A proteinase K accessibility assay was performed as previously described with slight modifications [16]. In total, 200 μL of cells (OD_600_ = 1.0) were centrifuged and re-suspended in 1 mL of proteinase K solution containing 15% (w/v) sucrose, 15 mM Tris-HCl (pH 7.8), 0.1 mM EDTA and 200 mg of proteinase K. The reactions were incubated at 37°C for 0.5 h and then immediately measure for whole-cell arginase activity used the Chinard reaction [17].

### Fluorescence detection with fluorescence microscopy and flow cytometry

All the antibodies were purchased from California Bioscience (USA). After washing three times with PBS, 200 μL of re-suspended cells (OD_600_ = 1.0) were blocked with PBSB [PBS (pH 7.4) containing 0.1% BSA] for 0.5 h, followed by three wash steps with PBS. Then, cells were incubated for 1 h at 4°C with mouse anti-HA tag antibody (1:1000 dilution in PBSB) followed by three washing steps with PBS. Then, cells were then incubated for 1 h with secondary antibody [FITC-conjugated goat anti-mouse secondary antibody, dilution of 1:1000 in PBSB] and secondary antibody[Dylight649-conjugated goat anti-mouse secondary antibody, dilution of 1:1000 in PBSB] respectively, followed by two washing steps with PBS. Finally, the antibody labeled cells were re-suspend in 0.5 mL PBS.

For the fluorescence microscopy observations, 7 μL of FITC-labeled samples were dipped onto a microscope slide and examined with a laser Zeiss LSM-710 confocal microscope (Jena, Germany) equipped with a 63×objective. Fluorophores were excited with an argon laser (488 nm), using the EGFP detecting channel.

For the flow cytometry assay, cells labeled with Dylight649 were analyzed using the Cytoflex cell sorter (Beckman Coulter, USA). Fluorophores were excited with the 638 nm laser, and emission signals were measured using the 660/20 BP filter.

### Characteristics of the engineered InaK-N/ARG1

The enzymatic activity of the surface-immobilized human ARG1 was assessed by the Chinard reaction [17], in which a reaction mixture containing 100 μL of L-Arg (0.2 M), 800 μL of bicarbonate buffer (50 mM, pH = 10) and 100 μL of each recombinant strain (OD_600_ = 1.0) was prepared. Reaction mixture was first pre-warmed for 5 min at 40°C in a water bath. Then, it was incubated at the same temperature with shaking for 10 min follow by centrifugation at 12,000 rpm for 2 min. The supernatant was collected and heated at 100°C for 5 min to terminate the reaction. The L-Orn content in the reaction buffer was detected using the Chinard colourimetric assay. In this study, one unit of enzyme activity was defined as the amount of enzyme required to produce 1 μM L-Orn/min at 40°C. The hydrolysis of L-Arg to L-Orn was also identified by MC trace (Agilent 6224 TOF, USA).

To optimize the temperature, cells were assessed in 0.05 M bicarbonate buffer at temperatures ranging from 30°C to 80°C. To optimize the pH, cells were assessed in phosphate buffer (50 mM, pH 6.0–7.0), Tris-HCl buffer (50 mM, pH 7.0–8.0), bicarbonate buffer (50 mM, pH 9.0–10.0) and hydrogen phosphate buffer (50 mM, pH 11.0–12.0) at 40°C. To optimize the metal ions, cells were evaluated individually in 0.05 M bicarbonate buffer with the addition of various metal ions at 40°C. All of the samples were collected and their residual activities were assessed as described above.

## Results

### Construction of engineered InaK-N and InaK-N/ARG1 vectors

The 537 bp *InaK-N* gene from *P*. *syringae* (KCTC1832) encoded a protein with a molecular weight of approximately 18.9-kDa. Two signal peptides and three charged polypeptides were anchored to the N-terminus of InaK-N (Fig 1A), generating the engineered Inak-Ns, which were termed as *InaK-N* (585bp), *ssMalE-InaK-N* (663bp), *ssTorA-InaK-N* (687bp), *D*_*6*_*-InaK-N* (603bp), *E*_*6*_*-InaK-N* (603bp), and *K*_*6*_*-InaK-N* (603bp) (Fig 1D). Human ARG1 was subsequently introduced into these newly generated constructs, which were termed *InaK-N/ARG1* (1548bp), *ssMalE-InaK-N/ARG1* (1626bp), *ssTorA-InaK-N/ARG1* (1650bp), *D*_*6*_*-InaK-N/ARG1* (1566bp), *E*_*6*_*-InaK-N/ARG1* (1566bp), and *K*_*6*_*-InaK-N/ARG1* (1566bp), respectively (Fig 1B and 1E).

### Confirmation of InaK-N anchors at the cell surface

**Fig 2:**
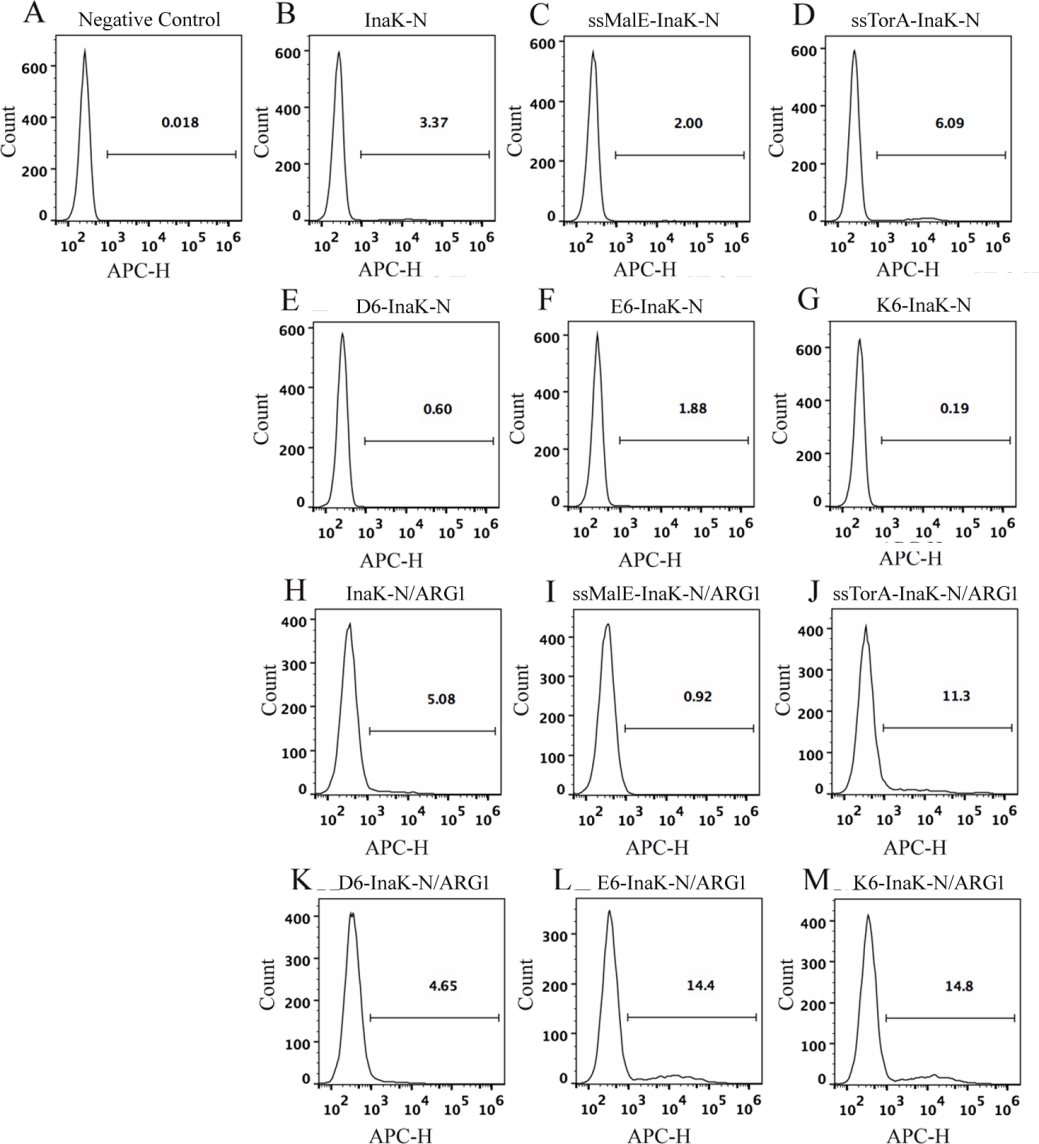
Flow cytometry assay for InaK-Ns and InaK-N/ARG1s. Cells containing different vectors labeled with Ddylight649-conjugated antibody against the HA epitope tag were analyzed by flow cytometry. The excitation laser was 638nm, and the emission filter was 660/20 BP. A-M indicated cells containing the pET23a-T empty vector; pET23a-InaK-N vector; pET23a *-*ssMalE-InaK-N vector; pET23a-ssTorA-InaK-N vector; pET23a-D_6_-InaK-N vector; pET23a-E_6_-InaK-N vector; pET23a-K_6_-InaK-N vector; pET23a-InaK-N/ARG1 vector; pET23a-ssMalE-InaK-N/ARG vector; pET23a-ssTorA-InaK-N/ARG1 vector; pET23a-D_6_-InaK-N/ARG1 vector; pET23a-E_6_-InaK-N/ARG1 vector; and pET23a-K_6_-InaK-N/ARG1 vector, respectively.

To quantitate the effect of the different polypeptides on the InaK-N surface display efficiencies, Dylight649-labeled cells were assessed by fluorescence-activated cell sorting (FACS) analysis (Fig 2, S1 Fig). In the control cells, only 0.018 ± 0.01% of the *Rosetta Blue* cells that contained empty pET23a-T construct presented strong Dylight649 fluorescent signal (Fig 2A), which indicated the low background due to the Dylight649-conjugated antibody. The cells containing the non-protein fused InaK-N displayed 3.37 ± 0.05% fluorescence (Fig 2B). Comparably, the Sec signal peptide decreased the InaK-N’s cell surface display efficiency by 41%, with only 2.00 ± 0.29% cells exhibiting fluorescence in the cells containing ssMalE-InaK-N (Fig 2C). However, InaK-N fused with the Tat signal peptide (ssTorA-InaK-N) showed enhanced cell surface display efficiency by approximately 2-fold, leading to 6.09 ± 0.15% of the total cells exhibiting fluorescence (Fig 2D). Our results also indicated that neither of the charged polypeptides, 6×Asp (D_6_-InaK-N), 6×Glu (E_6_-InaK-N), or 6×Lys (K_6_-InaK-N), could prompt InaK-N cell surface display efficiency, as only 0.19% ± 0.01% to 1.88% ± 0.06% of the total cells containing these constructs exhibited strong fluorescence (Fig 2E, 2F and 2G). The increased cell surface display efficiency caused by the N-terminal-fused TorA signal sequence indicated that InaK-N might fold in cytoplasm and then cross the inner membrane before anchoring in the outer cell surface.

### Confirmation of ARG1 fusion protein display on the cell surface

The modified InaK-Ns for cell surface display of the complicated foreign proteins were further evaluated. Human ARG1, which is a trimer in its active form, was not easily displayed on the cell surface using current bacterial surface display systems [18]. Therefore, the surface display of human ARG1 was evaluated with engineered InaK-N surface display systems (Fig 1B). Similar to the non-ARG1 fused InaK-Ns (Fig 2B–2G), cells containing ssMalE-InaK-N/ARG1 and D_6_-InaK-N/ARG1 presented lower fluorescent signals (Fig 2I and 2K) than cells containing InaK-N/ARG1 (Fig 2H). However, cells containing ssTorA-InaK-N/ARG1, E_6_-InaK-N/ARG1, and K_6_-InaK-N/ARG1 all presented higher Dylight649 fluorescent signals, indicating enhanced cell surface display (Fig 2J, 2L and 2M). Among the engineered InaK-Ns, K_6_-InaK-N/ARG1 exhibited the highest cell surface display efficiency with 14.8 ± 0.13% of the total cells labeled with anti-HA-Dylight649 antibody, which was 2.9-fold of InaK-N/ARG1 (5.08 ± 0.08%) (Fig 2H and 2M). It was interesting to note that 11.3 ± 0.33% of the cells containing pET-*ssTorA-InaK-N/ARG1* and 14.4 ± 0.04% of cells containing pET-*E*_*6*_*-InaK-N/ARG1* were fluorescently labeled (Fig 2J and 2L), indicating their ability to up-regulate the cell surface display ability of InaK-N/ARG1.

**Fig 3:**
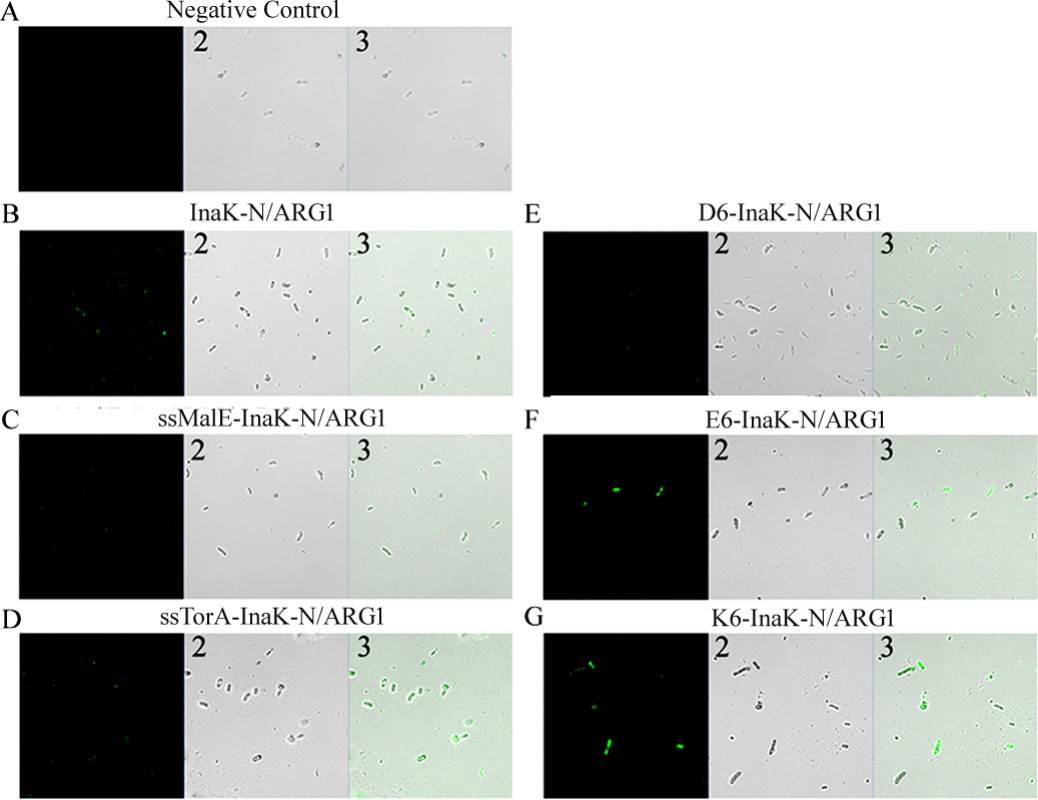
Fluorescence microscope assay of InaK-N/ARG1s. Surface fluorescence of the cells harboring various InaK-N/ARG1s under a fluorescence microscope with the excitation laser of 488nm, and the EGFP detection channel being used. A-G indicated cells containing empty pET23a-T vector; pET23a-InaK-N/ARG1 vector; pET23a-ssMalE-InaK-N/ARG1 vector; pET23a-ssTorA-InaK-N/ARG1 vector; pET23a-D_6_-InaK-N/ARG1 vector; pET23a-E_6_-InaK-N/ARG1 vector; and pET23a-K_6_-InaK-N/ARG1 vector, respectively. 1: detecting of FITC signal, 2: the bright field, 3: the merge of FITC signal and bright field.

The cell surface display of human ARG1 was further confirmed by immuno-fluorescence labeling of cells with a FITC fluorophore against the C-terminal HA epitope tag, followed by observation under the fluorescence microscope (Fig 3). As shown in Fig 3, solid fluorescence rods were observed in *E*. *coli* cells harboring ssTorA-InaK-N/ARG1, E_6_-InaK-N/ARG1, and K_6_-InaK-N/ARG1, indicating the presence of ARG1 fusion proteins on the surface (Fig 3D, 3F and 3G). In comparison, only weak fluorescence rods were observed in the *E*. *coli* cells harboring InaK-N/ARG1(Fig 3B), ssMalE-InaK-N/ARG1, and D_6_-InaK-N/ARG1, suggesting a lower cell surface displaying efficiency (Fig 3C and 3E). No fluorescence rods were detected in the control cells harboring the empty pET23a-T construct (Fig 3A).

**Fig 4:**
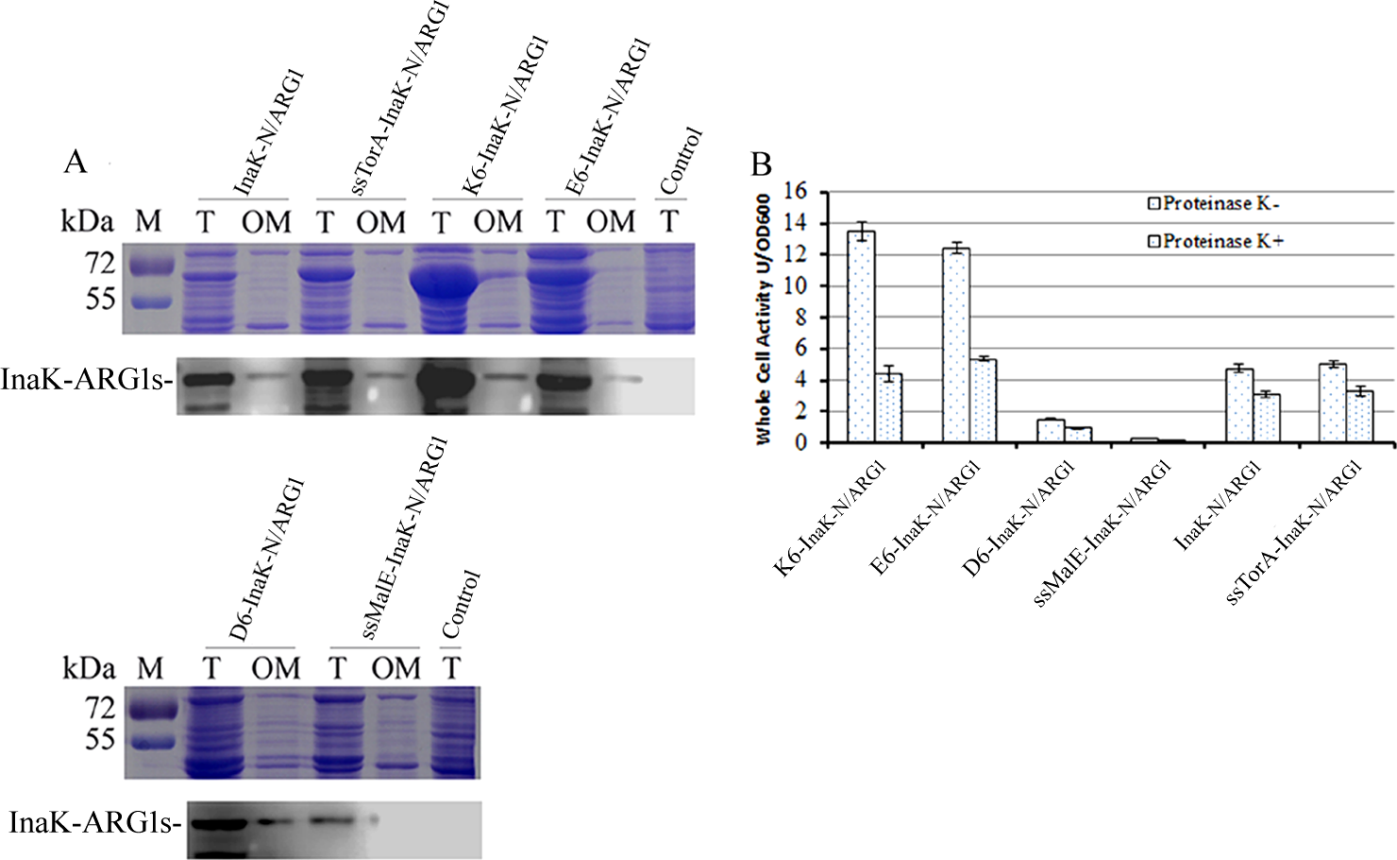
Immobilization of the ARG1 fusions to the cell outer membrane. (A) SDS-PAGE and Western-Blot analysis of the human ARG1 fusions on the outer membrane. Upper panel is the SDS-PAGE analysis and the lower panel is the western-blot analysis. M: protein ladder; T: total cell proteins; OM: outer membrane proteins. InaK-N/ARG1; ssTorA-InaK-N/ARG1; K_6_-InaK-N/ARG1; E_6_-InaK-N/ARG1; D_6_-InaK-N/ARG1; and ssMalE-InaK-N/ARG1 presented the recombinant strain expressing these fusion proteins, respectively; Control presented the recombinant strain containing empty pET23a-T. (B) Enzyme activity and proteinase K accessibility of the InaK-N/ARG1s. Proteinase K-: recombinant strain was untreated with proteinase K; Proteinase K+: recombinant strain was treated with proteinase K. K6-InaK-N/ARG1; E6-InaK-N/ARG1; D6-InaK-N/ARG1; ssMalE-InaK-N/ARG1; K6-InaK-N/ARG1 presented the recombinant strains expressing these fusion proteins, respectively.

We also fractionated the outer membrane proteins from the cells harboring the ARG1 fusion proteins, followed by analysis using SDS-PAGE and Western Blot (Fig 4A). The bands corresponding to each ARG1 fusion protein, including InaK-N/ARG1 (55.67 kDa), ssMalE-InaK-N/ARG1 (58.89 kDa), ssTorA-InaK-N/ARG1 (59.71 kDa), D_6_-InaK-N/ARG1 (56.91 kDa), E_6_-InaK-N/ARG1 (56.99 kDa), and K_6_-InaK-N/ARG1 (56.98 kDa), were all successfully detected in the total cell protein extracts as well as the outer membrane protein extracts (Fig 4A). Then, the surface-displayed InaK-N/ARG1s in the transformed *Rosetta Blue* cells were further confirmed by the proteinase K accessibility assay (Fig 4B and Table 2). Considering that proteinase K could only degrade the cell surface-presented proteins, the presence of the surface-displayed InaK-N/ARG1 proteins could be determined by analyzing the decrease in arginase activity from the whole cells. Not surprisingly, the proteinase K accessibility assay indicated a decrease in whole-cell arginase activity of approximately 35% for InaK-N/ARG1 (enzyme activity decreased from 4.75±0.23/OD_600_ to 3.10±0.19/OD_600_), 24% for ssMalE-InaK-N/ARG1 (enzyme activity decreased from 0.27±0.01/OD_600_ to 0.21±0.01/OD_600_), 34% for ssTorA-InaK-N/ARG1 (enzyme activity decreased from 4.99±0.21/OD_600_ to 3.31±0.28/OD_600_), 37% for D_6_-InaK-N/ARG1 (enzyme activity decreased from 1.49±0.06/OD_600_ to 0.95±0.04/OD_600_), 57% for E_6_-InaK-N/ARG1 (enzyme activity decreased from 12.43±0.38/OD_600_ to 5.34±0.17/OD_600_), and 68% for K_6_-InaK-N/ARG1 (enzyme activity decreased from 13.47±0.58/OD_600_ to 4.37±0.49/OD_600_) (Fig 4B and Table 2). Based on these results, it was confirmed that the fusion InaK-N/ARG1 was successfully anchored on the outer membrane of the *Rosetta Blue* cells, with K_6_-InaK-N/ARG1 allowing the highest proteinase K accessibility. This result was in accordance with the flow cytometry data that showed that K_6_-InaK-N/ARG1 displayed the highest cell surface display efficiency (Fig 2M).

**Table 2:** Whole cell enzyme activity.

Name	Proteinase K-(U/OD_600_±SD)	Proteinase K+(U/OD_600_±SD)
InaK-N/ARG1	4.75±0.23	3.10±0.19
ssMalE-InaK-N/ARG1	0.27±0.01	0.20±0.01
ssTorA-InaK-N/ARG1	4.99±0.21	3.31±0.28
D_6_-InaK-N/ARG1	1.49±0.06	0.95±0.04
E_6_-InaK-N/ARG1	12.43±0.38	5.34±0.17
K_6_-InaK-N/ARG1	13.47±0.58	4.37±0.49

### Characterization of the activity and stability of the surface-immobilized human ARG1 fusion proteins in *E*. *coli*

**Fig 5:**
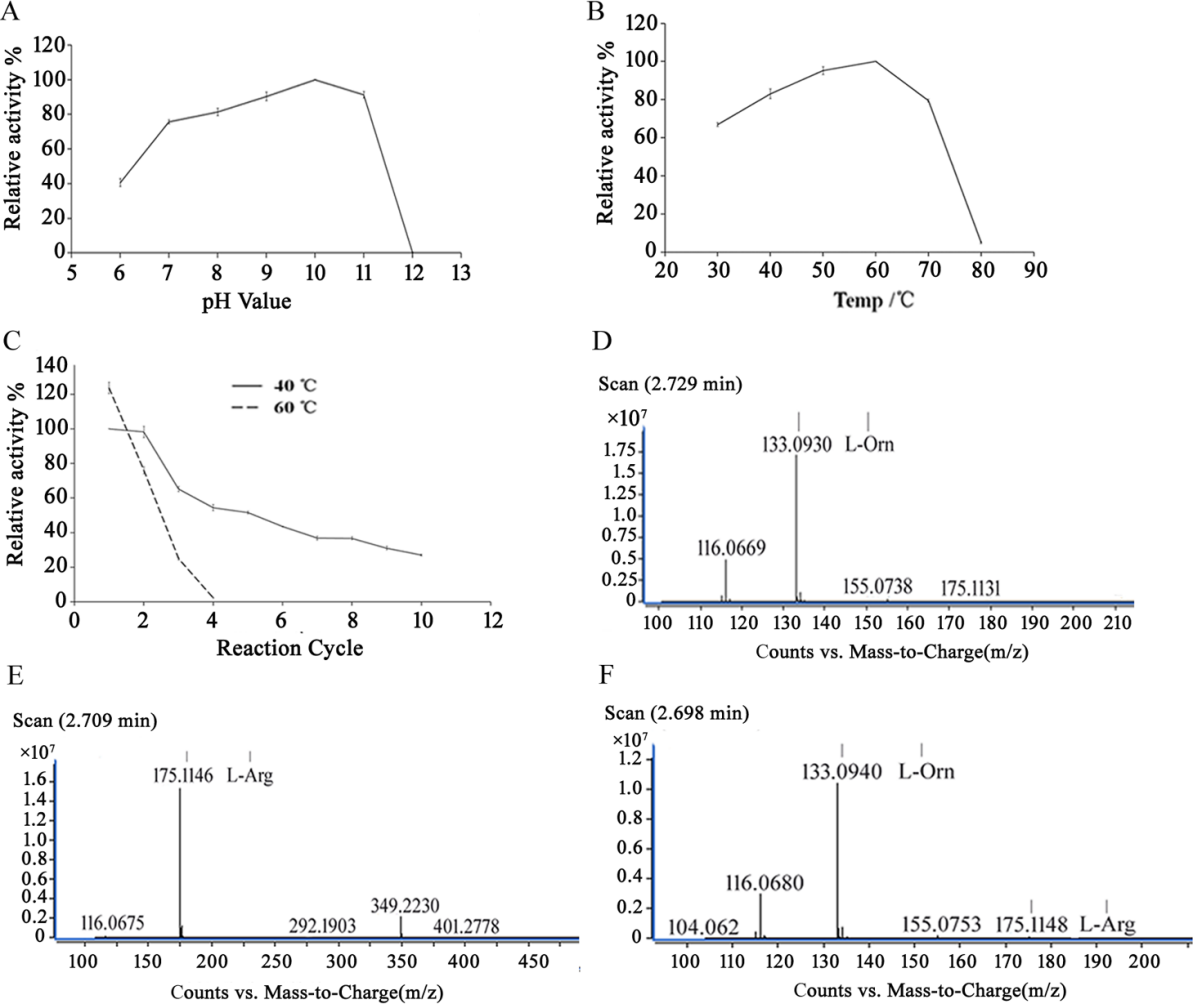
Characterization of the cell surface-immobilized InaK-N-E/ARG1s. (A) Relative activity of the cells bearing K_6_-InaK-N/ARG1 at different pH values. (B) Relative activity of the cells bearing K_6_-InaK-N/ARG1 at different temperatures. (C) Operational stability of the cells bearing K_6_-InaK-N/ARG1. The maximum activity was normalized to 100% in all of the assays. (D) MC trace analysis of the hydrolysis of molecular standard L-Orn. (E) MC trace analysis of the hydrolysis of molecular standard L-Arg.(F) MC trace analysis of the hydrolysis of L-Arg catalyzed by the cells bearing K_6_-InaK-N/ARG1.

Because K_6_-InaK-N/ARG1 had the best display efficiency and enzyme activity among the ARG1 fusion proteins, we further characterized the K_6_-InaK-N/ARG1 to optimize its L-Arg hydrolysis efficiency. It was determined that the optimum pH and temperature for the L-Arg hydrolysis reaction catalyzed by K_6_-InaK-N/ARG1 were pH10 and 60°C (Fig 5A and 5B). It was also interesting to note that metal irons were identified to have different effects on the enzyme activity. Co^2+^, Mn^2+^, Ni^2+^, Cd^2+^, and K^+^ barely affected enzyme activity, while Fe^3+^, Ca^2+^, Zn^2+^, and Fe^2+^ significantly decreased enzyme activity (Table 3).

**Table 3:** The chemical reagents used in this study.

Reagent	Concentration (mM)	Relative activity (%)
Control	0	100
Mn^2+^	2	97.30±0.51
Co^2+^	2	102.55±0.42
Fe^3+^	2	49.85±0.41
Ni^2+^	2	99.38±0.26
Cu^2+^	2	78.13±0.45
Ca^2+^	2	46.14±0.46
K^+^	2	98.76±0.14
Zn^2+^	2	57.73±0.43
Cd^2+^	2	93.35±0.37
Mg^2+^	2	80.29±0.38
Fe^2+^	2	22.10±1.77
EDTA	2	38.49±1.07

Surface-immobilized human ARG1 has many potential industrial applications, especially when the displayed K_6_-InaK-N/ARG1 also possesses high operational stability in a batch reactor. In our experiments, the results indicated that the activity remained at more than 40% of its initial value after 10 cycles at 40°C, pH 10, and 1 mM Mn^2+^. However, at 60°C, pH 10, and 1 mM Mn^2+^, the surface immobilized K_6_-InaK-N/ARG1 could only be used for 4 cycles (Fig 5C). The optimal reaction conditions were further confirmed by the MS measurements. It was determined that nearly 100% of the L-Arg (200 g/L) was hydrolyzed into L-Orn after 16 h by the surface immobilized K_6_-InaK-N/ARG1 at 40°C, pH 10, and 1 mM Mn^2+^. In the MS measurements, an almost non-detectable L-Arg signal indicated a nearly full conversion of L-Arg to L-Orn by the surface immobilized human ARG1 (Fig 5D, 5E and 5F).

## Discussion

INPs are located on the outer membrane surface of gram-negative bacteria. INPs identified from *Pseudomonas syringae*, *Erwinia herbicola*, *and Xanthomonas campestris* have been well-characterized [7, 19]. These proteins have a typical structure and consist of an N-terminal domain, a C-terminal domain, and a highly repetitive central domain [8]. Although various proteins of interest have been efficiently displayed on the surface of bacteria through the INP-mediated cell surface display system [8, 20], the molecular mechanism of this display system has remained unclear as no specific signal sequence being identified [4].

In this study, a truncated INP (InaK-N) that only contained the N-terminal region of INP was engineered for cell surface display of human ARG1. Our results indicated that the Tat secretion pathway signal peptide, ssTorA, could largely prompt the INP surface display system, which increased the surface display efficiency of InaK-N and InaK-N/ARG1 to 80.7% and 122.4%, respectively (Fig 2). Comparably, the Sec secretion pathway signal peptide, ssMalE, decreased the surface display efficiency of InaK-N and InaK-N/ARG1 to 40.7% and 81.9%, respectively (Fig 2). It is difficult to provide direct experimental evidence for protein folding status in live cells, especially under the circumstance that the surface display mechanism of INP is still unclear. It could be speculated that the ssTorA signal peptide might guide InaK-N through the Tat secretion pathway. Compared to Sec channels, the diameter of Tat channels is as large as 70 Å [21–22]. Therefore, it can accommodate larger proteins to facilitate the surface transportation of Tat signal peptide-guided InaK-N. Additionally, the Sec pathway transports unfold protein through the inner membrane with possible aggregation in the periplasmic space [23], while the Tat pathway can escort pre-folded proteins through the inner membrane with no aggregation [24]. The flow cytometry analysis and enzyme activity evaluation results demonstrated that InaK-N with the ssTorA signal peptide exhibited a better display efficiency and increased enzyme activity, while ssMalE signal peptide gave a decreased display efficiency and enzyme activity.

Considering that InaK-N could be well-folded in *E*.*coli* [23], a Tat signal peptide leading the transportation of InaK-N through the Tat secretion pathway would further prompt its surface translocation. However, this does not exclude the possibility that InaK-N was originally secreted through the Tat secretion pathway, in which ssTorA provides a typical and stronger Tat signal peptide to enhance the secretion. Based on our results here, although we still cannot confirm the secretion mechanism of InaK-N, it can be reasonably speculated that the transportation of folded InaK-N in the cytoplasm to *E*.*coli* surface was helped by the ssTorA, which might be related to the Tat pathway.

Based on the flow cytometry data, the display efficiency of InaK N/ARG1 and ssTorA-InaK N/ARG1 are 5.08% and 11.3%, respectively, indicating an approximately 2-fold increase of display efficiency of ssTorA-InaK-N/ARG1. Comparably, the (whole cell) enzyme activity assay only suggested around 8% enhancement of enzymatic activity of ssTorA-InaK-N/ARG1. We speculate that this might be because the extra ssTorA signal peptide might affect the structure of ARG1, causing lesser mono ARG1 forming into the functional trimetric structures. As we know, the ARG1 has to form an effective trimetric structure to carry out its enzymatic function. Any slight alterations of one ARG1 monomer in this trimetric structure might affect the final enzymatic activity.

Another interesting finding in our study was that charged polypeptides, including 6×Lys and 6×Glu, could significantly increase the surface display efficiency of InaK-N/ARG1 by approximately 2-fold, even though they almost abolished the surface translocation of InaK-N (Fig 2). In contrast, the 6×Asp polypeptide down-regulated the InaK-N display system (Fig 2E and 2K). These surprising effects of the charged polypeptides might be associated the N-terminal pI-specific directionality and their interactions with InaK-N or human ARG1. It was reported that the total translational efficiency of the proteins was based on the ΔG_RNA_ value of the N-terminal coding regions, which were crucial for promoting more efficient translocation through the Tat channel [24]. Moreover, a short N-terminal polypeptide with a correct pI value and hydrophilicity could substitute for the Tat signal sequence with improved efficiency [24]. In our study, when 6×Lys, 6×Glu, and 6×Asp were fused as leader peptides to the N-terminus of InaK-N, the display efficiency of the non-protein fused InaK-N decreased from 3.37% to 0.19% and from 1.88% to 0.60%, respectively. The pI values for K6-InaK-N, E6-InaK-N, and D6-InaK-N were calculated to be 8.52, 4.91, and 4.81, respectively, which were significantly different from the pI value of the original InaK-N of 5.80. The highly altered pI values of these engineered InaK-Ns might significantly change the surface display properties of InaK-N, leading to the decreased cell surface display efficiency. At the same time, the pI values of E_6_-InaK-N/ARG1 (pI 5.74) and K_6_-InaK-N/ARG1 (pI 7.58), which probably helped sustain the functional properties of InaK-N. More importantly, it is possible that 6×Lys and 6×Glu might prompt the solubility of human ARG1 in *E*. *coli*, therefore increasing the surface translocation of InaK-N/ARG1. The down-regulated effects of 6×Asp might be caused by its lower translocation efficiency compared to 6×Lys and 6×Glu [24]. Moreover, the direct interactions between 6×Lys and 6×Glu with human ARG1 might also contribute its enhanced cell surface display. To further understand the effect of charged polypeptides, we also investigated the effects of 6×Arg polypeptide to the surface display efficiency of InaK-N and InaK-N/ARG1. The flow cytometry results indicated that the surface display efficiency of R6-InaK-N was 3.6%, while that of R6-InaK-N/ARG1 was 2.6% (S2 Fig). Considering that the surface display efficiencies of InaK-N and InaK-N/ARG1 are 3.37% and 5.08%, respectively, we speculated that 6×Arg polypeptide down-regulated the InaK-N/ARG1 surface display efficiency. The molecular mechanism of how the charged polypeptides affecting the surface display of INP remains unclear.

Using the engineered InaK-N surface display system, human ARG1 was displayed on the cell surface; the K_6_-InaK-N/ARG1 presented the highest display efficiency with an arginase activity of 13.47 U/OD_600_. This cell surface-immobilized human ARG1 could convert nearly 100% L-Arg (200 g/L) to L-Orn in 16 hours under optimal condition of 40°C, pH 10.0 and 1 mM Mn^2+^ (Fig 5). The MTT cell growth assay was performed to evaluate the levels of cell death during the enzymatic reaction. According to the results, the cell concentration changed form 0.85×10^8^/mL before the reaction to 0.68×10^8^/mL after the reaction, which indicates about 20% cell death during the 16 hours ARG1 experiments. Although the cell death happened during the reaction process, the functional surface immobilized human ARG1 still could actively catalyze the conversion of L-Arg to L-Orn. To the best of our knowledge, the work presented here is the first report that human ARG1, which functions as a trimer, can be actively displayed on the surface of *E*. *coli* through the INP surface display system. By coupling with FACS technology, surface-immobilized ARG1 might provide an alternative method for engineering human ARG1 in a high-throughput manner.

In summary, the TorA signal peptide increased the surface translocation of InaK-N, suggesting a possible role for the Tat secretion pathway, instead of the Sec secretion pathway, in INP transportation in *E*. *coli*. Moreover, the charged polypeptides, 6×Lys and 6×Glu, significantly increased the cell surface display efficiency of human ARG1 by the InaK-N surface display system. The cell surface-immobilized human ARG1 displayed full activity for efficiently hydrolyzing L-Arg to L-Orn. Although the detailed molecular mechanism by which 6×Lys and 6×Glu displayed opposite regulatory effects against the InaK-Ns fused with or without human ARG1 is unclear, the results presented in our study provide new insights for displaying proteins of interest using the InaK-N display system.

## Supporting Information

S1 Fig Flow cytometry assay for cells under different culture conditions. Containing pET23a-Inak-N vectors were grown under different culture conditions, followed by labeling with Ddylight649-conjugated antibody or FITC-conjugated antibody against the HA epitope tag. The labeled cells were then analyzed by flow cytometry with the excitation laser of 638nm, and the emission filter of 660/20 BP.
(TIF)

S2 Fig Flow cytometry assay for R6-InaK-N and R6-InaK-N/ARG1. Cells containing different vectors were labeled with Ddylight649-conjugated antibody against the HA epitope tag, followed by being analyzed using flow cytometry. The excitation laser was 638nm, and the emission filter was 660/20 BP. A-C indicated cells containing the pET23a-T empty vector; pET23a-R6-InaK-N; and pET23a-*R*_*6*_-InaK-N/ARG1, respectively.
(TIF)
