# Supplementary figures and images for: Surface Immobilization of Human Arginase-1 with an Engineered Ice Nucleation Protein Display System in E. coli
Source: PLoS One. 2016 Aug 1;11(8):e0160367. doi: 10.1371/journal.pone.0160367 (PMC4968799; doi:10.1371/journal.pone.0160367)

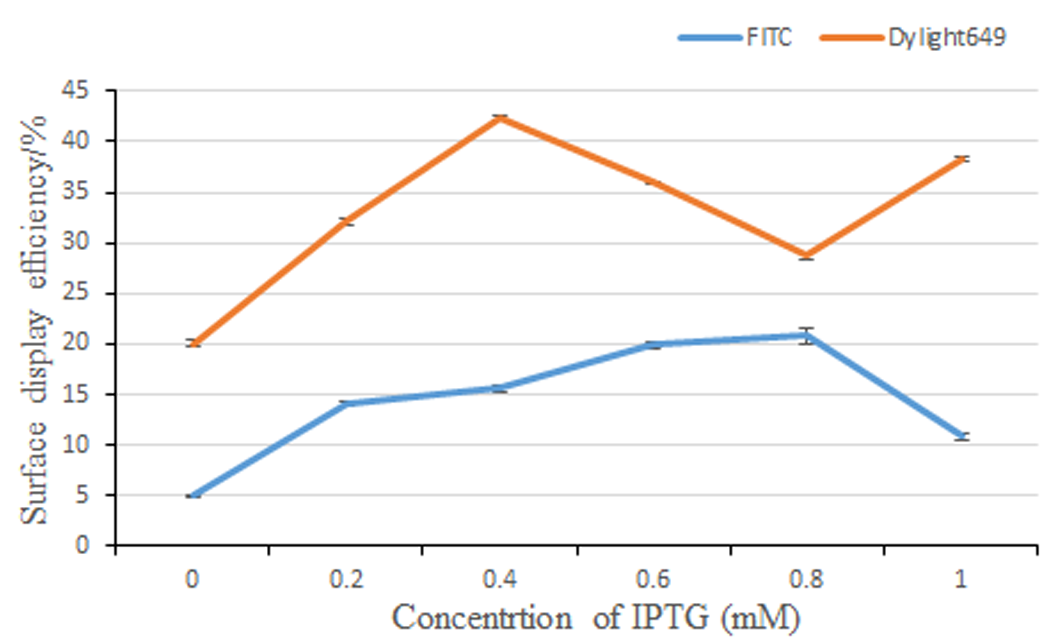

Supplement: S1 Fig — Containing pET23a-Inak-N vectors were grown under different culture conditions, followed by labeling with Ddylight649-conjugated antibody or FITC-conjugated antibody against the HA epitope tag. The labeled cells were then analyzed by flow cytometry with the excitation laser of 638nm, and the emission filter of 660/20 BP. (TIF) [file pone.0160367.s001.tif]

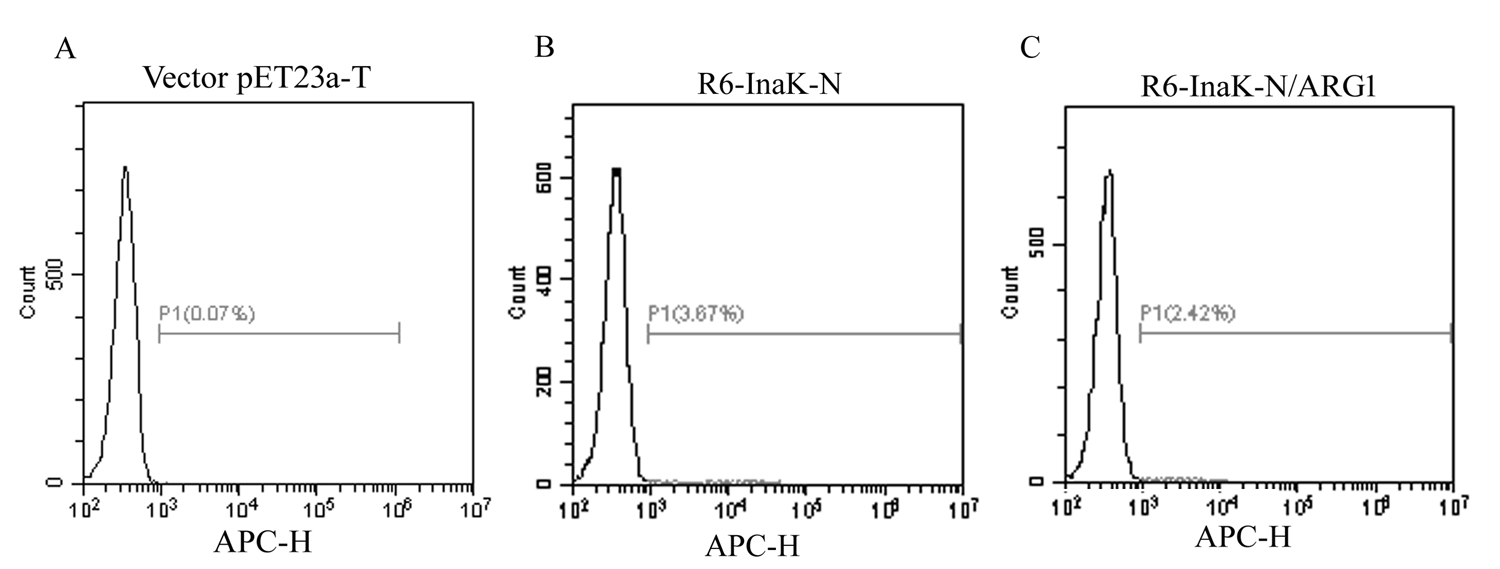

Supplement: S2 Fig — Cells containing different vectors were labeled with Ddylight649-conjugated antibody against the HA epitope tag, followed by being analyzed using flow cytometry. The excitation laser was 638nm, and the emission filter was 660/20 BP. A-C indicated cells containing the pET23a-T empty vector; pET23a-R6-InaK-N; and pET23a-R6-InaK-N/ARG1, respectively. (TIF) [file pone.0160367.s002.tif]
